# M-pick, a modularity-based method for OTU picking of 16S rRNA sequences

**DOI:** 10.1186/1471-2105-14-43

**Published:** 2013-02-07

**Authors:** Xiaoyu Wang, Jin Yao, Yijun Sun, Volker Mai

**Affiliations:** 1 Department of Epidemiology, College of Public Health and Health Professions and College of Medicine, Emerging Pathogens Institute, University of Florida, 32610, Gainesville, FL, USA; 2 Department of Electrical and Computer Engineering, University of Florida, 32610, Gainesville, FL, USA; 3 Department of Microbiology and Immunology, Center of Excellence in Bioinformatics & Life Sciences, Department of Computer Science and Engineering, The State University of New York at Buffalo, 14214, Buffalo, NY, USA

## Abstract

**Background:**

Binning 16S rRNA sequences into operational taxonomic units (OTUs) is an initial crucial step in analyzing large sequence datasets generated to determine microbial community compositions in various environments including that of the human gut. Various methods have been developed, but most suffer from either inaccuracies or from being unable to handle millions of sequences generated in current studies. Furthermore, existing binning methods usually require *a priori* decisions regarding binning parameters such as a distance level for defining an OTU.

**Results:**

We present a novel modularity-based approach (M-pick) to address the aforementioned problems. The new method utilizes ideas from community detection in graphs, where sequences are viewed as vertices on a weighted graph, each pair of sequences is connected by an imaginary edge, and the similarity of a pair of sequences represents the weight of the edge. M-pick first generates a graph based on pairwise sequence distances and then applies a modularity-based community detection technique on the graph to generate OTUs to capture the community structures in sequence data. To compare the performance of M-pick with that of existing methods, specifically CROP and ESPRIT-Tree, sequence data from different hypervariable regions of 16S rRNA were used and binning results were compared.

**Conclusions:**

A new modularity-based clustering method for OTU picking of 16S rRNA sequences is developed in this study. The algorithm does not require a predetermined cut-off level, and our simulation studies suggest that it is superior to existing methods that require specified distance levels to define OTUs. The source code is available at http://plaza.ufl.edu/xywang/Mpick.htm.

## Background

Recent advances in high-throughput sequencing technologies have contributed to an explosion in sequence data from studies of microbial composition in various environments that harbor complex microbial communities. As one of the most commonly used approaches for such studies, 16S rRNA sequences are analyzed to estimate species composition and diversity.

An initial requirement for downstream analyses of 16S rRNA sequences is the binning into operational taxonomic units (OTUs) that contain similar sequences. The existing methods can be divided into two classes, taxonomy-dependent methods and taxonomy-independent (TI) methods [1,2]. For taxonomy-dependent methods, query sequences are compared with known sequences deposited in annotated databases (e.g., RDP [3] and Greengenes [4]) [5]. Sequences that match with a reference sequence with a simialrity less than a predetermined cut-off value are grouped together. In contrast, TI methods apply clustering algorithms to pairwise sequence distances to assign query sequences into OTUs [6,7]. A major advantage of TI methods is their independence from the coverage of existing databases, which allows the analysis of sequences from unknown microorganisms, because novel sequences usually represent a large proportion of a sequence dataset [1].

In TI methods, pairwise sequence distances are computed either by multiple sequence alignment (MSA) or pairwise sequence alignment (PSA) and several clustering algorithms can then be applied to form OTUs. These clustering algorithms include hierarchical clustering algorithms such as DOTUR [8], MOTHUR [9], ESPRIT [7] and ESPRIT-Tree [10], as well as heuristic algorithms such as CD-HIT [6] and UCLUST [11]. In a recent benchmark study, we demonstrated that ESPRIT-tree appeared to have advantages in terms of both accuracy and computational efficiency [1].

One of the critical problems with existing TI methods is the need to set an appropriate distance threshold to retrieve the optimal OTU binning at a distinct taxonomic level such as species. Applying different thresholds leads to inconsistent binning results. Furthermore, appropriate distance levels appear to vary depending on the chosen hypervariable region [1], which makes it impossible to create one single distance-based threshold for defining a taxonomic level [2].

Some efforts have been made recently to address this issue. In [12], a semi-supervised clustering method was developed to identify a cut within a hierarchical clustering tree that maximizes the fit with a labeled subset of the sequences so that varied distance levels were applied in the clustering process to improve clustering accuracy. However, this approach shares a crucial disadvantage with taxonomy-dependent methods: the need to preselect labels to perform OTU picking. In [13], a Bayesian clustering method called CROP was developed, which uses a Gaussian mixture model to describe the pairwise sequence distance distribution in an OTU to avoid the need to set a single distance level for all clusters. Although this method does not use hard thresholds, it actually utilizes a lower and upper bounds that can be transformed to a threshold. Another Bayesian based method BEBaC [14] utilizes a crude 3-mer count based preclustering step, and then the partition space is searched for the partition having maximum posterior possibility for given sequence data. A minimum description length criterion is then applied in a fine clustering step to determine the number of OTUs and generate the final partitioning. Users only need to provide one parameter - the possible maximum number of OTUs as the input. The major disadvantage of this approach is its high computational cost.

In this study, a modularity-based clustering method was developed for OTU picking. By viewing an OTU as a collection of related sequences with similar densities in a sequence space, we applied a community detection method and treated OTU picking as a community structure detection problem.

## Methods

### Modularity-based clustering

We herein refer to community structure as the occurrence of groups of vertices in a graph that are more densely connected with each other than with the rest of the graph. Modularity-based methods are popular in community detection; they are derived from the intuition that a graph has community structure, if the number of edges within groups is significantly more than expected by chance [15,16]. Modularity *Q* of a partitioning result can be written as:

(1) $$Q=\frac{1}{2m}\sum_{\mathrm{ij}}\left(w_{\mathrm{ij}}-\frac{k_{i}k_{j}}{2m}\right)\delta \left(C_{i},C_{j}\right)$$

where *m* is the sum of weighted edges in the graph, is the weight of the edge connecting vertices and , is the degree of vertex *i*, i.e. the sum of weights on edges connected to vertex , and is the cluster that vertex is assigned to. The *δ* function represents the partitioning result information: if vertices *i* and *j* are grouped to the same cluster *δ*(*C*_*i*_, *C*_*j*_)=1, and otherwise *δ*(*C*_*i*_, *C*_*j*_)=0. The term

$$\frac{k_{i}k_{j}}{2m}$$

is used as the null model in Equation (1) to reflect the weight one can expect by chance [17].

Modularity itself is also a quality function that indicates whether a partitioning of a graph can reveal the community structure on the graph if such structure exists. The maximum value of modularity is 1; a large value implies good partitioning. The maximum *Q* value corresponds to the optimal partitioning on the graph, which best reflects its community structure. The community detection problem thus can be formulated as an optimization problem to find the partitioning that maximizes *Q*.

Several algorithms have been developed to efficiently optimize modularity. Among them, the algorithm in [18] appears superior in terms of both accuracy and speed [17,19], and it is chosen in our study to optimize modularity and find a clustering result that reflects community structure in our sequence data. The algorithm takes a bottom-up approach: it initially assigns each vertex to be a distinct cluster; it then moves a vertex into another cluster if the resultant modularity is increased; afterwards it recursively repeats the process by viewing each cluster as a vertex until a maximum modularity is obtained.

In the context of OTU picking, a weighted graph is formed by: i) viewing sequences as vertices, where each pair of sequences is connected by an imaginary edge, and ii) viewing the simlarity of a pair of sequences as the weight on the edge connecting these two sequences. Thus the modularity of a partition of sequences can be computed using Equation (1); the best clustering result is the one that maximizes the modularity. In such a result, each cluster represents an OTU with high homogeneity inside, that is, similarities between sequences within OTUs are greater than those between them. Using this approach, OTUs are defined by homogeneity of edge densities and not by distance between neighborhood clusters, circumventing the need for choosing distance levels.

**Figure 1:**
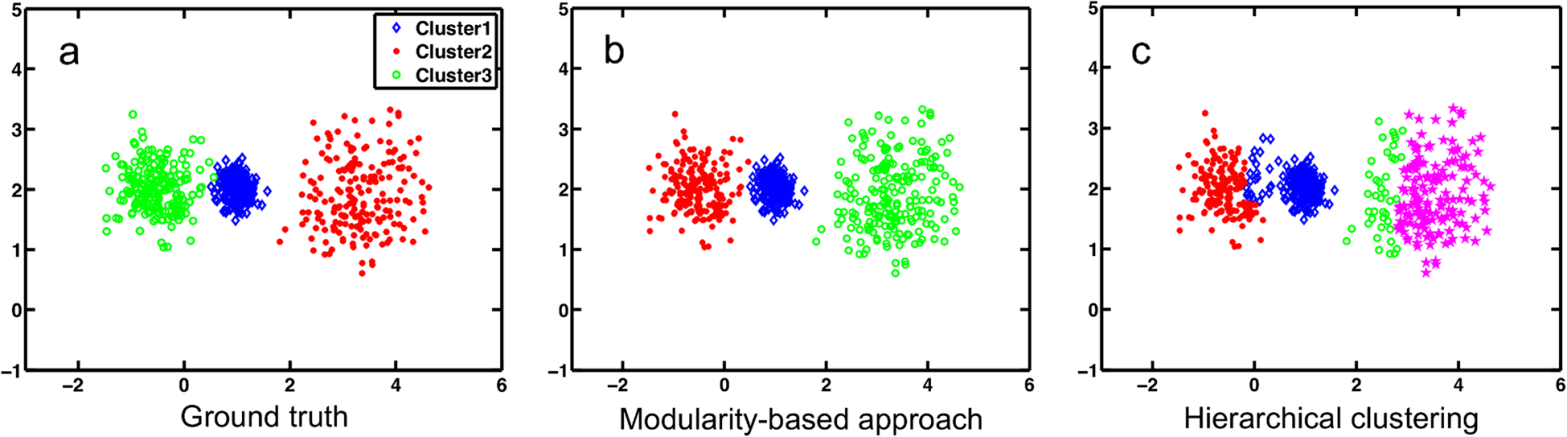
**M-pick outperforms hierarchical clustering when clusters have different sizes.** Clusters are represented in different colors. (**a**) Ground truth generated from three Gaussian distributions. (**b**) Clustering results of M-pick. (**c**) Clustering results of average linkage based hierarchical clustering.

A toy example comparing the modularity-based method and average linkage based hierarchical clustering is shown in Figure 1. The ground truth was generated from three Gaussian distributions with different means in x axis (-0.5, 1, and 3) and standard deviations (0.2, 0.4, and 0.6). The Euclidean distance is used to quantify the dissimilarity among vertices. There is no single distance level that effectively partitions these three clusters using hierarchical clustering; a variety of distance levels (0.05 to 3.5 with the increment of 0.05) have been applied in hierarchical clustering; its best result at distance level 2.80 is shown on Figure 1(c). In contrast, M-pick partitions the data properly when *ɛ*>=0.6 (see below) due to the fact that although clusters have different sizes, the vertex distances within a cluster are sufficiently smaller than those between clusters, and the density of weighted edges is higher within each group than that between groups.

Our modularity-based approach includes three steps. (1) Pairwise sequence distances are computed using the alignment module of ESPRIT [7]. (2) An *ɛ*-neighborhood graph is formed by only retaining the pairwise sequence distances less than *ɛ*, or equivalently pairwise sequence similarity greater than 1-*ɛ*. This step is somehow similar to single-linkage clustering. (3) Modularity-based clustering is recursively performed on the graph generated in the previous step.

In the first step, we generate a pairwise distance matrix, viewable as a fully connected graph. However, the fully connected graph cannot be directly used to perform clustering because of i) prohibitive computational costs and ii) the resolution limit problem which states that modularity-based methods may fail to acquire clusters smaller than a scale depending on the total size of the graph [20]. This implies that if a complete graph of significant size is used, small clusters in the graph will likely be ignored even if they show connectivity, albeit weak, to the rest of the graph and thus should be recognized as distinct OTUs. Therefore, we use a parameter *ɛ* in step 2 to mitigate these problems. Ideally, *ɛ* should be chosen to be greater than the maximum pairwise neighborhood sequence distance within a taxon, but not too large so that it includes all the sequences in multiple taxa into one connected graph. A graph formed in this way can guarantee that the sequences within a taxon are connected and the edge density within a taxon is greater than the density between taxa, making the community structure in the original fully connected graph more prominent.

**Figure 2:**
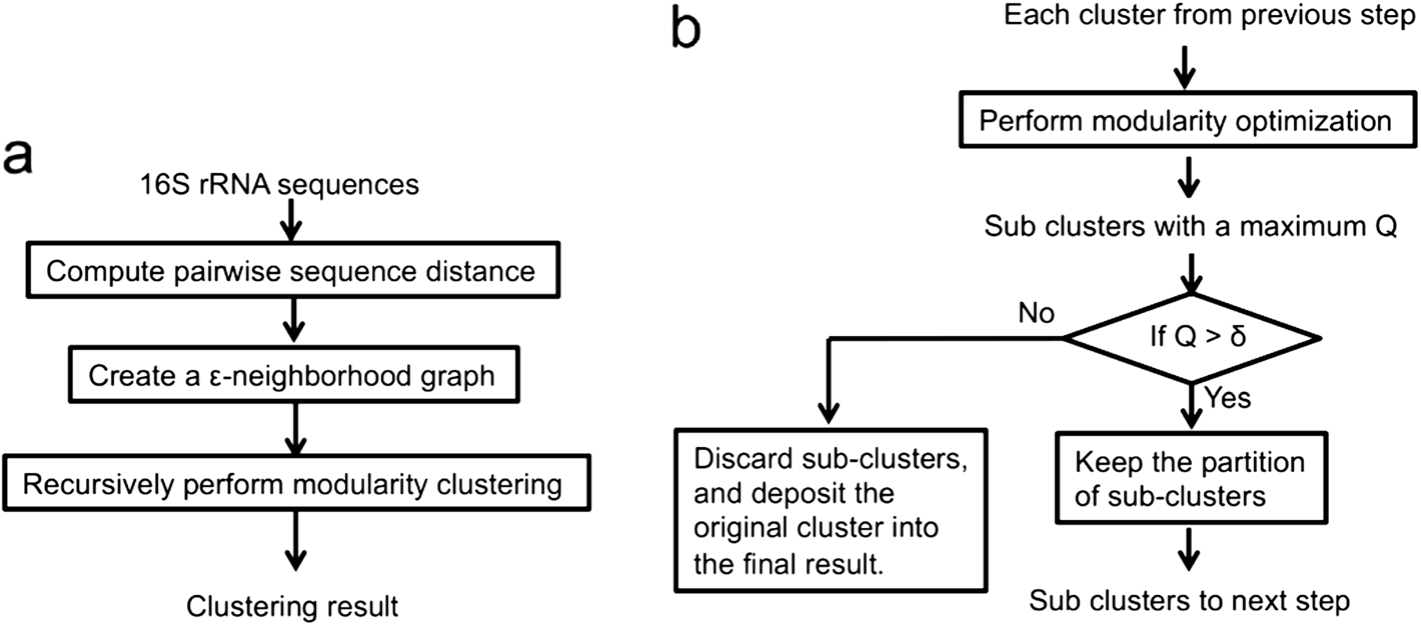
**Flowchart of M-pick.** (**a**) The overall process. (**b**) The recursive clustering process.

Due to the resolution limit problem, which often generates big clusters, it is not desirable to perform the clustering only once. Thus, we recursively evaluate each formed cluster to determine the need for further partitioning. The maximum modularity detected on a graph can indicate the presence of community structure in the graph. While a single cluster partitioning has modularity 0, partitions on a highly homogeneous graph (i.e., a graph with limited community structure) have modularity values close to 0. On the other hand, if multiple communities exist on a graph, some partitions will have large modularity values. Thus, the maximum modularity obtained on a graph can be used as a homogeneity criterion, suggesting the existence of multiple communities. Here we recursively apply clustering to sub-graphs exhibiting large modularity values, with the final sub-graphs or clusters having a maximum value less than a threshold *δ*. This recursive procedure - conducting modularity optimization on each single module is similar to that previously suggested by Fortunato et al. [20]. Our method is illustrated in Figure 2.

### Clustering results validation

Different clustering results are frequently obtained for the same sequence data set by applying different clustering methods and/or different parameter settings. The lack of a ground truth complicates an objective comparison of clustering methods. Generally, there are two types of clustering validation methods [21], either using external or internal criteria. Using external criteria the clustering results are compared to correct class labels from the 'ground truth', while only quantities inherent to the data are used for internal validation.

Normalized mutual information (NMI) is a well-known external criterion previously used for validating OTU picking; it measures the difference of a clustering result from a perceived ground truth [1]. NMI views the sequence distributions in the clustering result and ground truth as two discrete random variable distributions, and computes the NMI of the two random variables as the measure for quantifying agreement. The maximum NMI score is 1 which means the clustering result completely match with the partition in ground truth; the higher the NMI score, the more match. NMI is equivalent to variation of information used in White et al. [12].

Another popular external criterion is the F-score, which jointly considers precision and recall [22]. A common problem with F-score is that it does not satisfy the cluster completeness constrain that each cluster *ω*_*k*_ in ground truth is only judged by the best-matched cluster in the clustering result. Thus, other small clusters that match with *ω*_*k*_ can not affect the F-score, overestimating correlation when many small clusters are present [21,23].

Internal validation indices such as Silhoutette width [24] and Dunn index [25] have been used to evaluate clustering performance without the need for a ground truth. Quantities such as compactness, connectedness, and separation in the cluster distribution are used to evaluate clustering performance. While independence from questionable ground truths is a clear advantage, internal validation is only possible if the studied dataset has well-defined community structure, a condition that frequently is not met. For the above-mentioned reasons, we herein only use the external criteria based NMI score for clustering validation.

## Results

16S rRNA sequences of different hypervariable regions were used to compare M-pick with ESPRIT-Tree and CROP.

**Figure 3:**
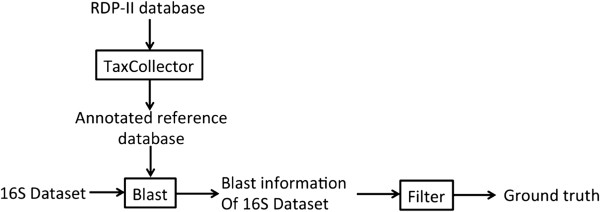
Procedures to generate ground truth for 16S datasets.

We first constructed a reference database from the RDP-II database [3], which was fully annotated using TaxCollector [26]. We then used various published 16S rRNA datasets of different hypervariable regions in our analysis. For each dataset, we ran a blast search against the reference database, and used a filter with the stringent criteria (>97% identity over an aligned region and >97% of the total length of the sequences) to retain the sequences that can be reliably annotated for use as the ground truth (Figure 3). 10 sub-datasets were then randomly picked from the retained sequences. The clustering algorithms were applied on these sub-datasets to compare their performances. A similar validation process has previously been described in detail [1,10].

### Case study 1 - V2 variable region

We used published sequences previously generated to study the association between obesity and the composition of human gut microbiota [27]. The dataset contains ~1.1M sequences covering the V2 region with an average length of 231 nucleotides. We blasted the sequences against the annotated RDP-II database, filtered the sequences using the criteria described in the previous section, and picked the species labels of the retained sequences as ground truth. We then randomly extracted 10 test subsets from these retained sequences, each containing 1000 sequences from the 50 most abundant species (total 50,000 sequences).

**Figure 4:**
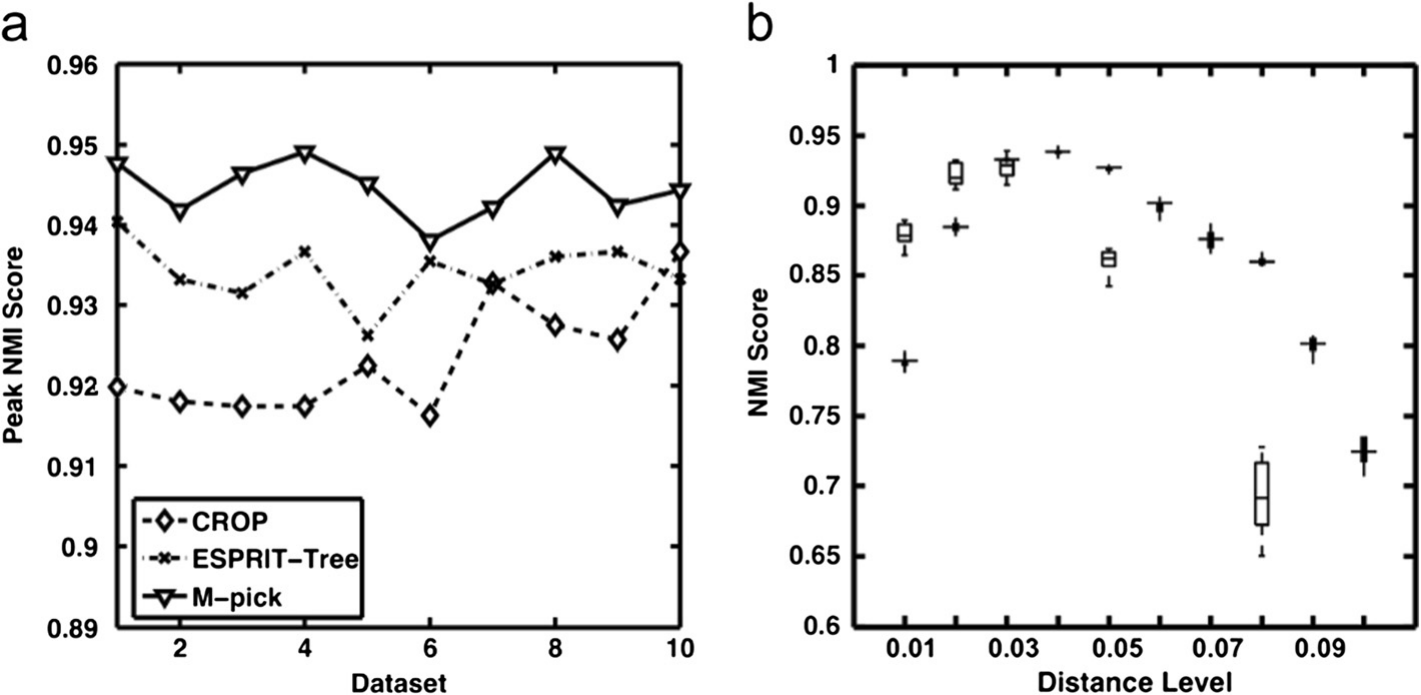
**Performance validation for Case study I.** (**a**) Peak NMI scores of CROP and ESPRIT-Tree compared with NMI scores of M-pick. (**b**) Boxplots of NMI scores of CROP (boxes, at cut-offs of 0.01, 0.02, 0.03, 0.05, and 0.08), ESPRIT-Tree (filled boxes, at cut-offs ranging from 0.01 to 0.1 incremented by 0.01).

ESPRIT-Tree was applied to each test subset using distance levels between 0.01-0.1 (incremented by 0.01) and the peak NMI score was chosen. Similarly, CROP was applied to each test dataset using different cut-off settings (1%, 2%, 3%, 5%, and 8%) as described in [13] and its peak NMI score was selected. M-pick was applied using a setting *ɛ* = 0.04 to generate a graph for each test dataset. 0.04 was chosen because for most cases it is greater than the distance between two sequences in a species in our ground truth. Thus, once we form the *ɛ*-neighborhood graph, sequences in a species are more likely to connect to each other than connect to sequences in other species and the edge densities of sequences within a species are generally greater than the edge densities of sequences from different species, which makes it appropriate to apply a modularity-based method. The stopping criterion for recursive clustering was chosen as *δ* = 0.1. The NMIs of the M-pick were compared with the peak NMIs from CROP and ESPRIT-Tree (Figure 4a). For illustrative purposes, the NMI scores of CROP and ESPRIT-Tree at different distance levels are shown in (Figure 4b).

While ESPRIT-Tree and CROP can achieve NMI scores greater than 0.9 at their optimum distance level, results are sensitive to the chosen distance level (which is not known *a priori*). M-pick generated the most accurate results for all of the test datasets.

In addition to the NMI scores, we also checked if the three methods could accurately estimate the number of species in the test datasets (Table 1). The estimations from CROP and ESPRIT-Tree were based on clustering results using their best distance levels. ESPRIT-Tree performed slightly better than the other two methods. As for standard deviations, M-pick generated the most robust estimations; its results were more consistent in all the test cases. It should be emphasized that the OTU number estimates from CROP and ESPRIT-Tree are all based on their optimal distance levels, which in real applications are unknown. M-pick can accurately estimate the number of species in test datasets without a need to specify a distance level for defining OTUs.

**Table 1:** Number of OTUs and the best distance levels of clustering algorithms (Case study 1)

	**CROP**	**ESPRIT-Tree**	**M-pick**
# OTU (mean, std)	55.5 (19.5)	45.3 (10.8)	56.6 (3.1)
Best distance level	2%–3%	4%–5%	N/A

**Figure 5:**
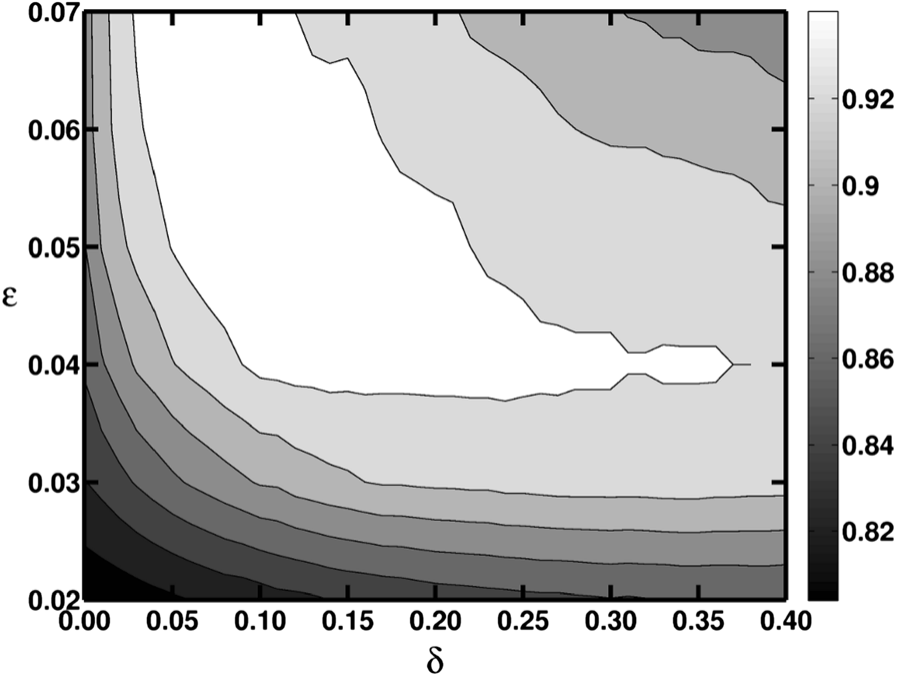
**NMI scores of M-pick using different ε and *δ *values in Case study 1.**

In order to evaluate the impact of parameter selection (*δ* and *ɛ*) on M-pick clustering results, we performed a simulation study (Figure 5). Parameters values within the area marked in white yielded more accurate results than the best result obtained using ESPRIT-Tree. Our simulation shows that M-pick performed very well over a wide range of parameters. However, if *δ* was too small (e.g., <0.03), it led to many small spurious OTUs. On the other hand, a large *δ* (e.g., >0.37) resulted in underestimation of the number of species by generating large OTUs. In both instances the NMI scores can be worse than the peak NMI scores of ESPRIT-Tree. As for *ɛ*, it should be greater than 0.038 (*ɛ*=0.038 is horizontally tangential to bottom of the white area). *ɛ* was selected as 0.04 in this case study partly due to the fact that in this case *δ* can be chosen in a broad region (0.09-0.37) in the white area so that it is more robust against *δ*.

### Case study 2- V9 variable region

To confirm the observation described above and to be able to generalize our findings, we performed additional studies using different datasets covering various 16S rRNA hypervariable regions. Results from another case study are presented below; The second study was performed on a dataset retrieved from a soil microbial diversity study [28] where 139,000 bacterial 16S rRNA sequences (hypervariable V9 region) were obtained from samples collected in Brazil, Florida, Illinois, and Canada.

**Figure 6:**
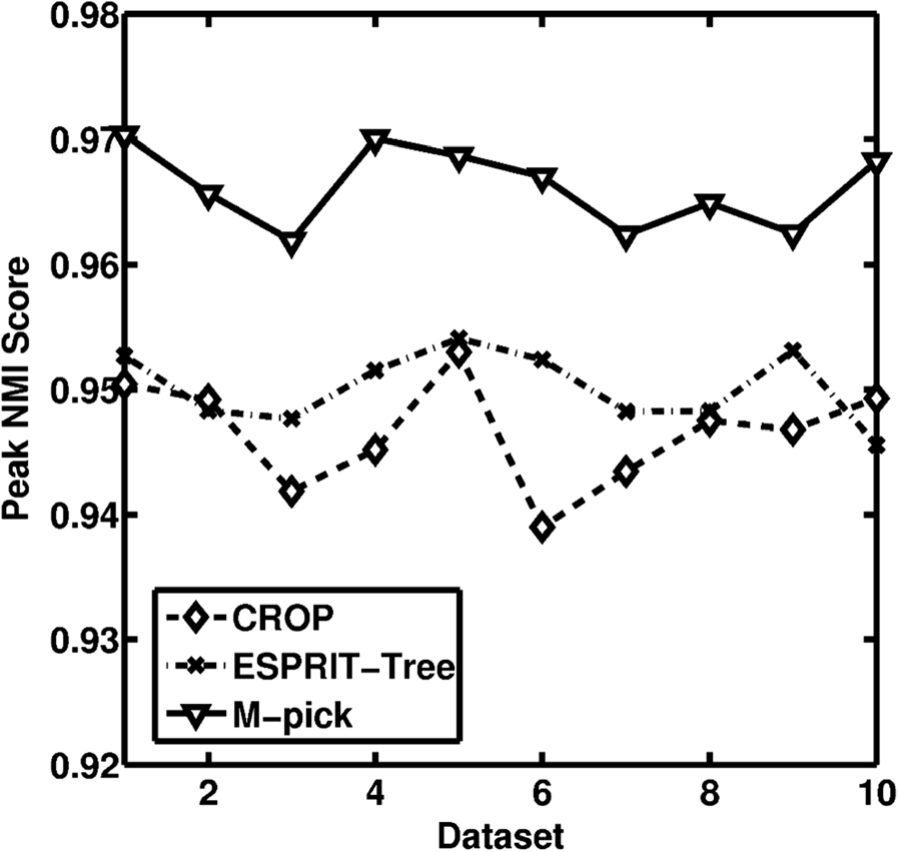
Peak NMI scores of CROP and ESPRIT-Tree compared with NMI scores of M-pick.

**Figure 7:**
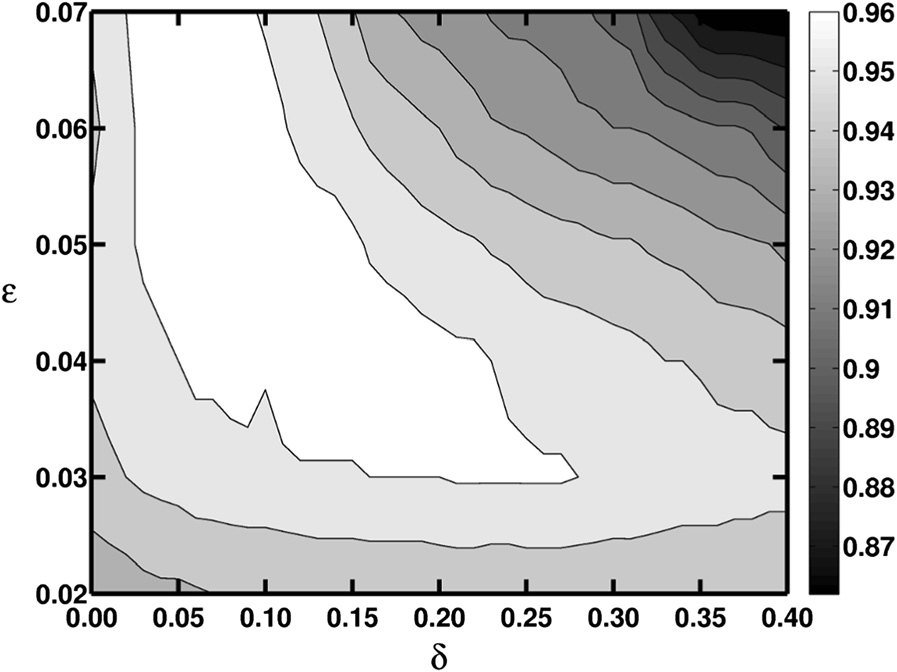
**NMI scores of M-pick using different ε and *δ *values in Case study 2.**

Similar to the first case study, we initially performed a blast search of the sequences against the annotated RDP-II database and filtered the sequences using the previously described criteria. We then randomly extracted 10 test subsets each containing 1000 sequences from the 100 most abundant species in the ground truth. The proposed M-pick algorithm was applied by setting *ɛ* = 0.04 to create a graph, and the stopping criterion was chosen as *δ* = 0.15, which is within the appropriate range depicted in Figure 5. CROP and ESPRIT-Tree were again applied to the test datasets and their peak NMI score compared with M-pick (Figure 6). Similar to the first case study, M-pick significantly outperformed ESPRIT-Tree and CROP in both accuracy and robustness. We also found that M-pick was superior to the other two algorithms when using a wide range of parameter settings, shown as the white area in Figure 7.

### Case study 3- V3 variable region

For the ease of presentation, we only used the top 50 or 100 species in the previous case studies, which may not give a complete picture of how M-pick works on a whole real data.

In this case study, we used a dataset from our sepsis study designed to investigate the association of sepsis and intestinal microbiota in infants with very low birth weight. The dataset contains 110,000 sequences from V3 region. ESPRIT-Tree and M-pick were applied to obtain clustering results for the whole dataset. *ɛ*=0.04 and *δ*=0.1 were used in M-pick. Afterwards, we blasted the dataset against the RDP-II reference database and applied the stringent filter to retrain a subset of 101,000 sequences that have species annotation. We then extracted the clustering result of the annotated sequences from the whole clustering results, and compared it with the species labels to validate the clustering performance. Again, we used the NMI score to compare M-pick and ESPRIT-Tree evaluated at different distance levels. The estimated numbers of OTUs and NMI scores were listed in Table 2. It can be seen that M-pick generated fewer number of OTUs but at the same time a higher NMI score, which implies that sequences belong to a species are more likely to be grouped together into the same OTU by using M-pick.

**Table 2:** Number of OTUs and NMI generated by ESPRIT-Tree at varied distance levels and M-pick (Case study 3)

	**ESPRIT-Tree**				**M-pick**
	**0.01**	**0.02**	**0.03**	**0.04**	
# OTUs	8823	2338	1356	944	921
NMI	0.846	0.870	0.859	0.831	0.879

### Case study 4- simulated dataset

In the above case studies, the ground truth was generated by keeping the sequences that highly matched with the RDP-II database through the stringent criteria. However, the way to genererate ground truth could be quenstionable. To adress this concern, we included another simulated dataset from [14], which contains 22,000 sequences from 11 taxa generated from a Gaussian distribution model with varied deviations. We applied M-pick on the data, and it correctly grouped sequences into 11 taxa with a perfect NMI score of 1, which is better than those from BEBaC, UCLUST, ESPRIT-Tree, and CROP shown in [14]. We also investigated how the problem of resolution limit affected the clustering results by keeping only 20 sequences from Taxon 8. M-pick still retrieved the correct clustering result, which confirms that M-pick worked well for this rare taxon case without the problem of resolution limit.

### Additional case studies

Additional case studies were provided in the Additional file 1. The results were consistent with the findings presented in the previous sections.

## Discussion

We herein developed a novel modularity-based clustering method, M-pick, for binning 16S rRNA sequences into OTUs. M-pick is based on graph partitioning, and does not require a predetermined distance level to generate OTUs, which is a challenging requirement for many other OTU picking methods.

M-pick is based on a concept from graph partitioning. It initially creates a similarity based graph composed of all the sequences in a dataset. The algorithm first computes the pairwise sequence distances, and then implicitly creates an *ɛ*-neighborhood graph from the fully connected graph by only keeping sequence connections with pairwise distances less than *ɛ*. This strategy is used to save computational cost and to make community structure in the original graph more prominent. Modularity is used not only as the quality function to perform clustering but also as the criterion for terminating the recursive clustering process. We stop partitioning a graph (cluster) when all of its partitions have a modularity value smaller than *δ*. Both settings of *ɛ* and *δ* help to alleviate the resolution limit problem. Although we cannot claim that the proposed method has solved the problem, we found in our empirical studies that the resolution limit does not seem to be a serious issue.

We used multiple sequence datasets from different hypervariable regions of 16S rRNA to compare the performance of M-pick with two other commonly used algorithms, CROP and ESPRIT-Tree. Both are thought to generate accurate clustering results if the optimal distance level is known. However, the optimal distance level, which is not known *a priori*, varies for different hypervariable regions and even for different datasets from the same region. M-pick outperformed the other two algorithms in most cases even when the optimal distance level was used in the two competing algorithms.

Two parameters are required by M-pick. *ɛ* is used in the process of creating a graph and *δ* is used to decide when to stop the recursive clustering. The constraint on an OTU introduced by *ɛ*and *δ* is different from that of preset distance level used in ESPRIT-Tree and CROP. It can create arbitrarily shaped OTUs, which alleviates the problem of similar sequences being split into separate OTUs. We found that *ɛ* should be chosen to be larger than the maximum pairwise neighborhood sequence distance within a species. In all datasets that we analyzed, *ɛ* and *δ* were set to be 0.04 and 0.1, respectively, and the results were superior to those achieved by the other two algorithms. Thus, we suggest users to use this parameter setting for picking OTUs at species level. A systematic study to determine the two parameters for other phylogenetic levels needs to be carried out in the future. For the stopping criterion *δ*, similar considerations should be taken as in [29] in order to determine this parameter based on the statistical significance of the maximum modularity values of sub-clusters generated in the recursive clustering process.

The computational cost is composed of two parts. (1) *O*(*n*^2^) is consumed in computing pairwise sequence, where *n* is the number of sequences. (2) The cost of performing modularity-based clustering is approximately linear with respect to *m*, the number of edges in an *ɛ*-neighborhood graph. The running time is mainly consumed in the computation of pairwise sequence distances. Therefore, it is highly desirable to develop a more efficient pairwise sequence alignment method. At present, large datasets are handled by adding a preprocessing step. Sequences are pre-clustered at 1% distance level using a high-speed method such as UCLUST, and a representative sequence from each cluster is used to form a reduced dataset, on which the pairwise sequence distances are computed.

## Conclusions

We developed M-pick, a new modularity-based clustering method, for OTU picking of 16S rRNA sequences. The algorithm does not require a predetermined cut-off value, and our simulation studies suggest that it is superior to the methods that require specified distance levels to define OTUs. M-pick appears to offer a viable alternative for binning similar sequences into OTUs.

## Competing interests

The authors declare that they have no competing interests.

## Authors’ contributions

XW, YS and VM designed the study. XW and JY performed the simulations. All authors discussed the results, read and approved the manuscript.

## Supplementary Material

Additional file 1 Results of case studies not included in the main text. Click here for file
