# Supplementary material for: M-pick, a modularity-based method for OTU picking of 16S rRNA sequences
Source: BMC Bioinformatics. 2013 Feb 7;14:43. doi: 10.1186/1471-2105-14-43 (PMC3599145; doi:10.1186/1471-2105-14-43)
Supplement: Additional file 1 — Results of case studies not included in the main text. [file 1471-2105-14-43-S1.pdf]

Case study on V6 dataset

We also validated our approach using a well-isolated dataset from [Huse07], which consisted of 340,000 V6 sequences amplified from 43 templates, being at least 3% different from each other.

We used the 43 template sequences as a reference database, and blast all the sequences against this database. The same filter (>97% identity over an aligned region and >97% of the total length of the sequences) was applied to the blast results to retrieve 320,000 sequences in the ground truth. All the clustering methods were applied on the retained dataset to compare their performances.

The computational cost for clustering is not too expensive as we often choose ε as small as 0.04, so only part of sequence distances need to be considered. However, it is still time consuming for large datasets to compute the pairwise distances. To remedy it, we first grouped the sequences at a small distance level (0.01) using ESPRIT-Tree and obtained 4118 OTUs, and we picked a representative sequence from each OTU to represent this OTU. We then applied M-pick on these representative sequences to get the clustering result for this sub dataset. M-pick was applied by setting $\varepsilon = 0.04$ in creating graphs to partition, and the stopping criterion was chosen as $\delta = 0.1$. Finally the result was used to retrieve the clustering results of the original whole dataset.

The clustering results are illustrated in Table 1. It is found that NMI scores of all the methods are all very high (>0.99) for this well-isolated dataset. Notice that the ground truth was created not in the same way as in [Hao11] which used a hierarchical clustering method (ESPRIT) on the 43 template sequences and used the number of obtained OTU to be the ground truth.

Table 1 Results of clustering algorithms (V6 dataset). The number of clusters in ground truth is 43.

	CROP	ESPRIT-Tree	M-pick
#OTUs	45	48	44
Best distance level	5%	5%	NA
Peak NMI	0.9989	0.9994	0.9998

Case study on full-length sequences

In addition to partial 16S rRNA sequences, we also conducted experiments on full-length sequences. The dataset contains 9920 near full-length sequences from [Turnbaugh09].

We blast these sequences against the annotated RDP database. The same filter (>97% identity over an aligned region and >97% of the total length of the sequences) was applied to the blast result to form the ground truth. We then randomly extracted 10 test subsets from the retained sequences, each containing 1000 sequences. M-pick was applied by setting $\varepsilon = 0.04$ in creating graphs to partition, and the stopping criterion was chosen as $\delta = 0.1$. CROP and ESPRIT-Tree were also applied to the test datasets. The NMI scores of M-pick method were compared with the peak NMI scores of CROP and ESPRIT-Tree, and the results are shown in Figure 1. It was found in this case the clustering

results of M-pick are comparable to the best results of ESPRIT-Tree (p=0.5576); probably it is due to the fact that full-length sequences allow finer discrimination than partial sequences so that if the optimal distance level is given, ESPRIT-Tree can work as good as M-pick.

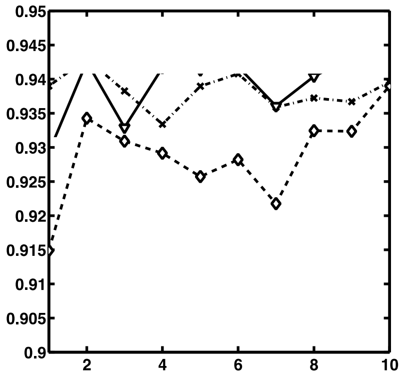

Figure 1 Peak NMI scores of CROP and ESPRIT-Tree comparing with NMI scores of M-pick.
